# Reconceptualizing the ethics of diagnosis: a Foucauldian framework

**DOI:** 10.1186/s13010-026-00227-0

**Published:** 2026-08-03

**Authors:** Antonia Sahm

**Affiliations:** 1 Professur für Ethik in der Medizin, University of Erlangen-Nuremberg, Erlangen, Germany; 2 Institut für Philosophie, Goethe - Universität, Frankfurt, Germany

**Keywords:** Medical diagnosis, Medical ethics, Michel Foucault, Social epistemology

## Abstract

**Background:**

Traditional approaches in medical ethics have primarily focused on the consequences of diagnoses, emphasizing their impact on patients’ lives, treatment options, and social perception. While this perspective remains indispensable, it is limited in its consideration of the conditions under which diagnostic categories are created and applied. However, understanding these processes is essential, as they shape not only individual patient experiences but also broader social norms and the framework conditions of health systems.

**Methods:**

Drawing on Michel Foucault’s work, this paper examines the intersection between epistemic authority and ethics in the production of medical knowledge. Foucault’s analyses of medicine reveal how diagnostic practices are not purely objective but are embedded in institutional frameworks that regulate the production and application of medical knowledge. This perspective resonates with recent discussions in medical ethics such as epistemic injustice. However, as these often converge with Foucault’s ideas, his work provides a suitable basis for a comprehensive examination of the way in which diagnoses are handled.

**Results:**

Diagnoses are not merely neutral descriptions of objective biological phenomena, but emerge from specific epistemic and institutional frameworks that structure medical knowledge. A Foucauldian lens allows for the formulation of critical questions that encourage a shift in perspective, from evaluating only the consequences of diagnoses to interrogating the processes that produce and validate them. This approach highlights the ethical significance of medical epistemology itself and its influence on patient care, social norms, and health governance.

**Conclusions:**

Ethical analysis in medicine must extend beyond the immediate effects of diagnosis. Attention to the production and structuring of medical knowledge is necessary to fully understand how diagnoses influence patients’ experiences, shape social expectations, and reinforce systems of power. With the help of a Foucauldian Shift, medical ethics can more comprehensively address the moral dimensions of diagnosis, moving from consequences to the broader conditions that generate them.

## Introduction

### Diagnosis as a turning point

Diagnosis is a central step in the medical process. It is integrated between anamnesis, examination and therapy and is the pivotal node in the medical process. Without diagnosis - that is, the identification and understanding of a condition - causal therapy cannot be initiated, medical therapies would be arbitrary [6]. Diagnosis, therefore, serves as both a crossroads and a turning point in medical processes. Emerging from suffering and investigation, it represents the recognition of an illness and, at best, allows for a reversal of its course. Ideally, a correct diagnosis leads to a therapy that cures the condition. At a minimum, the purpose of diagnosing is to alleviate suffering. Whether it is a tentative diagnosis, a confirmed diagnosis, or a differential diagnosis, it lays the foundation for determining therapeutic indications and provides orientation.

However, diagnosis is not merely a decisive step in the medical process in the sense of a causal basis of biomedical procedures. It also opens up another dimension, one that cannot be fully captured or defined by biomedical categories alone. This dimension pertains to the social aspects embedded in diagnoses when applied to individuals. The social impact of diagnoses affects individuals in their daily lives and self-perception. This social and psychological dimension has long been a subject of sociology and ethical research, as diagnoses can exert power over individuals and influence medical processes such as therapy compliance. This becomes apparent, for example, when a diagnosis associated with social stigma leads individuals to reject it, causing them to refuse therapy or avoid seeking help altogether [38, 51].

As early as 1844, Harrison pointed to the influence diagnoses can have on patients with his remark: *“Diagnosis can be more dangerous than the disease”* [28]. Diagnoses harbor a tension that continues to engage social medicine and ethical research today. Jutel articulated it as follows:

Diagnosis “is an explanatory moment in which a group of symptoms may be given meaning and context. It is also a predictive moment at which a prognosis is often projected; even non-terminal diagnoses have strong structuring impact on the social reality of an individual.” [31] “It gives rise to ‘bucket lists’ and religious conversions; it is the subject of memoirs and of creative writing. The description of the diagnostic moment infuses poetry and prose and presents an important symbolic moment in the drama of the doctor-patient relationship.” [31]

The fact that diagnoses are not solely biomedical categories but also carry social significance necessitates closer examination. Exploring the impact of diagnoses on patients, and their social environment is essential to address the ethical dimensions of these formally biomedical categories.

This article addresses the interaction between biomedical knowledge, as practised in Western medicine, and social contexts with the aim of contributing to a framework for the ethics of diagnosis. Medical ethics itself is a constitutive practice. It shapes expectations of medicine and influences medical practice. Recognising the power of medical-ethical discourse entails maintaining critical engagement with its focus, assumptions, and practices. The paper first outlines how medical ethics discourse conceptualizes diagnosis and presents a structural analysis of this perspective. The established view is then expanded through a Foucauldian lens, which highlights the emergence and use of social diagnoses shaped by underlying social assumptions and judgments. This approach reveals the complex dynamics between biomedical and social dimensions of diagnosis. Finally, the article demonstrates the relevance of a Foucauldian turn for medical-ethical debate by raising questions and considerations for applied ethics. Building on this approach, it argues that medical ethics must critically interrogate the normative frameworks and epistemic premises underlying the production of medical knowledge in order to develop arguments that are both comprehensive and compelling.

## Social implications of diagnoses and intervention approaches

### Social implications of diagnoses

Diagnoses can impact individuals and their ways of living as soon as they are suspected, and particularly once they are articulated by experts, documented in writing, and coded using Diagnosis Related Groups (DRGs). Suffering becomes a reality for the individual through medical recognition. What was previously an individual feeling transforms into a medical symptom and cannot easily be dismissed, especially because it is validated by others and, more specifically, by the institutional authority of the physician. The feeling, once personal and vague, becomes part of a universally accessible category. From then on, it becomes comparable and intelligible to others. It can be evaluated and commented on by the social environment. This transformation, from an individual experience into an objectified biomedical category, is a significant shift brought about by diagnoses. To enable medical action, initiate therapies, and achieve healing or alleviation of suffering, this objectification of suffering is necessary. The experience must become intelligible. At the same time, this objectification has different effects on the lives of patients.

On the one hand it can be a relief, while on the other, it can significantly burden a person. Relief can arise when patients realize that they are not alone in their suffering and that others have taken on the task of helping them. Through diagnosis, responsibility is shared [32]. In this way a diagnosis can lead to increased attention and care, which patients may find comforting. Beyond that it can be comforting to discover that there is an objective category into which one’s suffering fits. The experience of having one’s suffering described in an objectifiable way can lead to it being considered valid and the suffering being deemed legitimate. Recognition by others is often crucial. Conversely, a lack of recognition of suffering or an inability to name it can result in loneliness and self-doubt [33]. For example, a sense of distress can be observed when symptoms remain unexplained, and no diagnosis can be established [15]. A diagnosis may provide patients with the recognition that their experiences are collectively shared and that established frameworks for coping are available. Only through a diagnosis can the prospect of ending or alleviating the suffering emerge. The diagnosis is the foundation for determining the indication to begin therapy.

However, relief is not all that is felt when a diagnosis is given. Being diagnosed with serious illnesses can be extremely distressing, and assumptions and prejudices associated with certain illnesses can have an impact on the individual. This risks categorising conditions in a way that is tainted by prejudice. The diagnosis of a mental illness, for example, might be associated with madness, danger to self and others or lack of accountability [4, 56]. The diagnosis of a tumor might evoke anxiety and the horror scenario of imminent death [8, 39, 52] while diagnoses of obesity, diabetes or hypertension can be associated with blame and an unhealthy lifestyle [29, 41, 55]. This can result in great shame among those affected. These are just a few examples of how diagnoses can intersect with social assumptions and prejudices. Overall the stigma that patients carry can lead to social exclusion and isolation. Due to stigmatization, some patients may avoid therapies, exhibit non-compliance, or attempt to conceal or ignore their illness [47]. Thus fear of a diagnosis can lead individuals to avoid visiting doctors and seeking medical help, because “the (diagnostic) utterance marks a boundary. It serves to divide a life into “before” and “after,” and this division is henceforth superimposed onto every rewrite of the individual’s life story“ [16].

### Approaches addressing the implications of diagnoses

As outlined above, biomedical categories, when applied in medicine, often intersect with social categories, sometimes to the detriment of patients. This can manifest in forms such as stigma and prejudice associated with certain illnesses. Medical ethics seeks to address this by employing both quantitative and qualitative methods to identify the effects that diagnoses have on individuals. For example, it examines whether the naming of specific conditions leads to increased isolation or whether certain psychological diagnoses carry additional societal burdens [26, 35, 45, 46].

Furthermore, medical ethics dedicates attention to the specific impacts of diagnoses e.g. by offering psychological counseling to oncology patients or including patients’ close social circles in educational efforts about diagnoses [5]. These measures aim to familiarize the patient’s close environment with the diagnosis and reduce potential alienation. Developing strategies to counteract the stigmatization of various illnesses is therefore a key focus within medical ethics, emphasizing the social dimensions of diagnoses.

Other ethical and sociological approaches addressing the social aspects of diagnoses focus directly on the patient. These approaches aim to empower patients, giving them greater control over the medical process and decision-making. By ensuring they understand the rationale behind a diagnosis, patients can anticipate and evaluate the therapeutic procedures and their effects on daily life. Here, the impact of the diagnosis on the patient’s everyday existence is specifically considered. The prominent concepts of Shared- Decision-Making (SDM) [13, 53] and Informed Consent (IC) [34] are two of many examples of such an approach. They empower patients to choose between different treatment options or to decline treatments altogether. The focus on patient autonomy is another discourse that at least implicitly recognises the social impact of diagnoses. These ethical considerations highlight that the social consequences of diagnoses significantly influence therapeutic decisions. Factors such as the patient’s living conditions affect therapeutic outcomes, underscoring the need to tailor treatment concepts to these circumstances and ensure they are feasible for the patient.

While these concepts are not explicitly aimed at addressing the social dimensions of diagnoses, they serve to empower patients. In doing so, the social and bodily experience of patients is considered and becomes a critical factor in shaping subsequent medical procedures. Efforts to promote patient autonomy in therapeutic processes help create spaces where the social consequences of diagnoses can be shaped in the best interests of the patients. This way ethical discussions around effective medical decision-making aim to design the social implications of diagnoses in ways that prevent patients from being passively subjected to them. The overlap between social and biomedical categories continues to be one of the central challenges of medical ethics and is therefore widely discussed, both explicitly and implicitly.

Overall, the focus of medical ethics lies on the impact and consequences of biomedical categories on the social lives of patients. The reason for this is, that negative effects are immediately evident in patients’ lived experiences and can have significant repercussions on the patient´s psyches, and everyday realities. Recognising these connections is a relevant field of work in medical ethics and deserves attention. However, in order to grasp the connection between biomedical diagnostic categories and their social dimension, another perspective can be useful. With the help of Michel Foucault’s work, an expanded perspective on this dynamic can be achieved. Although Foucault’s work has been applied to medical contexts, it occupies only a marginal position in medical-ethical debates. Revisiting and reactivating his perspective, however, offers considerable potential and should be integrated into medical ethics as a critical lens for more comprehensive ethical reflection.The Foucauldian shift makes it possible not only to look at the social impact of diagnoses, but also to consider the extent to which values and social assumptions influence diagnostic categories and allows to question the application of these categories in their complexity.

## The Foucauldian shift

### Key conceptions of Foucault's work

As outlined, there is a strong awareness that diagnoses are not merely biomedical categories but also carry significance beyond clinical contexts. In ethical reflections, the focus is on reducing the negative social and psychological effects that diagnosis can have on patients. However this focus could suggest a neutrality of biomedical categories, which are only imbued with values, ideas, and attributes - such as those leading to stigmatization - when applied to patients. This relationship and interaction between biomedical categories and social assumptions are further explored through the work of Michel Foucault who opens up a different view. In his writings, Foucault highlights how values are not only something imposed on biomedical categories after application as diagnoses. He shows how values, assumptions and political objections shape the way research is conducted and knowledge is formed. This way he points out that social and political problems can serve as starting points for the production of knowledge. His method is discourse analysis and genealogy which demonstrate that what is considered true or scientific is contingent on specific frameworks and subject to change over the course of history: showing that different priorities have been emphasized at different times [19, 20, 22, 23].

With this inquiry and methodology, Foucault’s perspective challenges the assumption of biomedical categories as neutral entities that acquire values and attributes only upon application [7]. His work underscores that biomedical categories and research are shaped by the social conditions in which they are created, pointing to an epistemic conditionality [3, 22, 23]. From a Foucauldian perspective, it is not only biomedical data that gets transformed into social categories. Norms influence how data is collected and how research is conducted. In his works he repeatedly emphasises the significance of this understanding for medicine. This perspective will be explored and applied to medical-ethical considerations in the context of diagnosis through a sketch of three key concepts from his work. These are *regimes of truth* [21] *biopower* [18] and the *clinical gaze* [19, 20] These three terms help to define a field where Foucault’s contribution to an ethical debate becomes clear.

When Foucault introduces the concept of regimes of truth, he highlights a connection between power and truth. Knowledge and power form a network that can mutually reinforce each other [21–23]. He points out, that on the one hand, that what comes to light as truth is contingent upon the conditions prevailing at a given time. This means that certain forms of knowledge are weighted according to the recognition that these knowledge practices receive in the social space. On the other hand, it also points to the fact that these truths can be instrumentalized to pursue various goals. This perspective means that truths are not neutral in their application, but can be used functionally: they can be instrumentalised to legitimise political, economic or social goals. Truth becomes a technology of power, a tool with which subjects can be controlled and societies regulated [17, 18, 24]. This perspective is central to modern medical ethics, as it highlights that medical decisions—such as those concerning diagnoses, treatment options, or resource allocation—are not determined solely by facts, but also by normative assumptions that often remain implicit.

Foucault specifies this in his later works in relation to medicine with the help of the concepts of biopower. With this term, Foucault refers to the interaction of (especially medical) knowledge with social contexts and the use of knowledge for the benefit of certain political goals, e.g. a strong regulation and optimisation of health services. This goes hand in hand with the control of biomedical processes and the statistical recording of the population’s health behaviour. It results in production of knowledge about bodies and medical data about groups that influence political decisions. This constellation harbours the risk of misuse. The danger, which Foucault points out and which is central to ethical considerations, is that knowledge can be instrumentalised, i.e. the generation of knowledge can be a technology of power [17, 18, 24]. In this context, medicine becomes a central locus of biopolitical control: knowledge about the body is used to establish behavioural norms, identify risk groups, compile statistics and legitimise state health measures. This practice serves a regulatory function– with the aim of optimising the health of the population, but also of establishing control.

The two concepts presented become practically effective for the individual in medicine through the clinical gaze. In The Birth of the Clinic he introduces the clinical gaze and describes the human being, who is the subject of medicine, as a place of representation and as the embodiment of society’s needs [19, 20]. Although the concept is introduced in his early work, when considered within the broader scope of his thought, it complements the other two concepts. The clinical gaze, which leads to diagnoses based on biomedical categories, is embedded in circumstances that enable this specific way of perceiving the body. It marks a specific epistemic configuration in which the body is no longer understood as a metaphysical or symbolic entity, but becomes an analysable object of medical examination. The clinical gaze is based on certain institutional arrangements and linguistic conditions: only that which can be named can be observed. The hospital or clinic functions as a structural apparatus that enables and maintains this type of observation. In this way, people and their bodies are objectified to the outside world. However, the seemingly neutral scientific description is conditioned by the language available to it [19, 20]. Against the background of regimes of truth and biopower, the clinical gaze serves as an instrument of these power constellations and proves to be an interface where power and knowledge meet in the context of medicine. Accordingly, the ostensibly neutral, scientific depiction of the body is inseparable from the power relations within which it is produced, serving instead as a manifestation of those relations. This way the clinical gaze is not merely an epistemic act but a power-knowledge *dispositif*, wherein epistemic authority and political interests converge. Foucault elucidates not only that knowledge is inseparable from power, but also how power is inscribed upon the body, thereby stabilizing societal norms, disciplinary mechanisms, and broader structures of governance through corporeal regulation.

In this connection of the three concepts, the mechanisms through which bodies are disciplined in medical contexts become visible. Patients are subjected to the clinical gaze and, with it, to medical categorization. The clinical gaze thus serves as an instrument of disciplinary power, when it is capable of influencing behaviour. This creates an apparent opposition in which physicians, on the one hand, and patients, on the other, seem to occupy distinct positions within this power relation. However, when the clinical gaze is considered within the broader context of the regime of truth, it becomes evident that physicians themselves are disciplined through the exercise of their expertise. The apparent localization of this power in the person of the physician arises from the restriction of individual agency through the attribution and enactment of expertise according to established rules and procedures, thereby securing the recognition of authority [48]. The categorizing function of the clinical gaze therefore does not originate in the physician’s gaze itself, but rather in the conditions that determine what can appear within that gaze in the first place.

The three keywords presented here are intended to outline Foucault’s perspective and serve as a starting point for focussing on the emergence of diagnoses and biomedical categories. All three concepts point to the connection between power and knowledge. The clinical gaze serves as an executive instrument on the individual, complementing structural observations as a general ‘regime of truth’ on the one hand and medically charged ‘biopower’ focused on the population on the other. This triad is intended to serve as a sketch of the perspective to guide the Foucauldian shift in the ethical view on diagnoses.

### Contextualisation and significance of the Foucauldian view

In order to apply these core ideas of Foucault’s work presented here, it is useful to situate his position within existing debates in medical theory and to clarify the interpretation of this position, by also addressing criticism. A return to Foucault can be helpful, as his work serves as the foundation for many contemporary theories that critically interrogate the ostensibly neutral and objective description of disease. For example, in the context of discussions on Epistemic Injustice and Social Epistemology similar questions have been raised, that point e.g. to the connection between power and language [1, 25]. Both currents, however, are united by their reliance on fundamental Foucauldian ideas and concepts. His scholarship, which both underpins these contemporary discussions and was developed in the context of medicine, offers a precise and pointed way to illustrate this shift for ethics while engaging with current debates.

Does Foucault propose a normative theory of disease and health? Foucault´s work resists clear categorization. Instead, it builds on the self-evident premise that “both medical practice and lay thought shape disease concepts” [40]. Foucault animates this idea in a constructive manner, offering significant insights for philosophy, the social sciences, and medical ethics [54] His work goes beyond the binary dispute between normative and naturalist models of illness [10] functioning instead as a threshold within this dichotomy—or more precisely, as an avenue into a new dimension. It is not necessarily fruitful to frame an examination of Foucauldian theses from this perspective. By shifting the focus to the tangible conditions under which medicine is practiced, he intertwines the dynamics of power with the production of knowledge in an accessible manner revealing their inherent complexity without falling into relativism of truth. The primary focus is not on defining what constitutes a disease or on accurately describing its characteristics, but rather on examining how diseases are managed. What real-world conditions influence the treatment of diseases and the practice of medicine, thereby impacting both sick and healthy individuals? What inherent risks of instrumentalization and misuse arise within these frameworks? In this way, Foucault touches upon the debate of normative and naturalistic concepts of health [7, 43] and may contribute to peripheral deliberations. However, his position cannot be clearly situated within this broad dichotomy, nor were his efforts aimed at adopting a stance within this or a similar line of debate [44]. Rooted in this application-oriented orientation, Foucault´s theory offers profound value for applied ethics.

To carry out this application of Foucault’s perspective in the following, it is also essential to examine the critique of his concepts in order to clearly elaborate on how these concepts can be attribute to ethics and what significance such a shift may have. So does his perspective imply that truths are relative if they are influenced by social conditions? And how can Foucault´s viewpoint be fruitful for medicine and medical ethics?

As already indicated above, adopting a Foucauldian approach does not necessitate embracing relativism, nor does it require the rejection of empirical evidence and scientifically grounded knowledge. Such criticisms would fail to do justice to Foucault’s work and overlook its potential. Foucault does not negate scientifically established truths or findings [19]. Instead, he invites an examinaton of these truths in their genesis and within their contextual frameworks [2]. He offers the opportunity to question when and under what conditions we submit to certain truths and grant them validity [37]. By doing so, it becomes possible to identify factors that influence the objectivity of these findings and to incorporate such reflections into scientific disciplines, such as medicine, and their diagnostic categories. In this way, Foucault helps to gain a clearer understanding of the biomedical categories commonly employed in medical practice, highlighting both their potential and their risks. He also calls for the identification of areas in which these categories may be instrumentalized for political purposes and provides tools to critically address and investigate such forms of instrumentalization. Contrary to criticisms that accuse Foucault of relativism or denial of truth [27, 30], his approach can serve as a means to identify the conditions that pave the way for *alternative truths* to challenge scientific findings. Both the glorification of scientific truths as immutable, eternal certainties and the outright rejection of claims to truth would be equally problematic [58]. The history of medicine, in particular, has frequently revealed its own errors in hindsight. Thus, an ethical engagement with scientific and biomedical categories is essential - one that acknowledges the potential of these categories, uphold the scientific credibility while remaining transparent about their risks and susceptibility to error. This way the Foucauldian shift represents a perspective that permits an examination of biomedical categories in the context of diagnoses. It can serve as a tool for clarifying and making these dynamics transparent, thereby laying the groundwork for an ethical approach to diagnoses.

## Foucault and the ethics of diagnosis: emphasizing the ‘Before’

The following sections will illustrate Foucault’s reflections through selected examples, with the aim of clarifying their pertinence to contemporary contexts. These examples focus on the stage preceding the diagnosis of the sick individual. This includes an exploration of the emergence of biomedical categories and their inherent blind spots, as well as an analysis of the social impact of diagnoses prior to their application, particularly in the context of preventive measures.

### The emergence of biomedical categories

The significance of Foucault’s work for medical-ethical considerations lies in the fact that it focuses on the factors and circumstances that influence the development and emergence of diagnostic categories. It allows not only to consider the *after* of the diagnostic moment – the application. Foucault’s perspective in contrast allows to focus on the *before*, removing the illusion of considering this phase before the application of diagnosis on the individual as shielded from the social dimension. This emphasizes the importance of examining the research questions and identifying the contextual conditions in which diagnostic categories arise. With the help of Foucault’s concept of regimes of truth and biopower the connection between truth and power can be revealed – before and after the diagnostic moment.

By shifting the focus to what precedes the diagnostic moment, the influence of political and social interests and values becomes clear. These are shaped by historical events and production conditions, as theorized by Foucault in his account of regimes of truth and the interrelation of power and knowledge. Such framework conditions reorient the focus of research, the clinical perspective, and public attention towards specific interests. Biomedical research is not immune to these influences. Importantly, they are not merely assumptions, opinions, or values; they have a material component with direct and visible impact. On the one hand, this is most evident in the economic interests that influence medical research and lead to certain diagnoses. On the other hand, it raises the question of which research questions are pushed into the background and become an epistemic blind spot as a result.

Economic interests influence the framework conditions and research questions of biomedical research. Public interest and demand for therapies are the driving forces behind investment in treatments. Establishing suffering as a diagnostic category is crucial as it paves the way for further research and enables financial investment in research to develop therapies. Only what is perceived as suffering and recognised as a health problem can be classified as a diagnostic category and treated. Diagnoses such as Gender Dysphoria or Long-COVID are examples of this dependency. They have appeared as forms of suffering, but the interpretation of this suffering was not solely left to biomedical research; it has also entered the political domain. There, debates unfold over whether these diagnoses should be recognized as valid diagnostic categories or whether they should be subsumed as symptoms under other mental health conditions. Such a decision influences whether the diagnosis is recognized and, consequently, whether investments are made in specific therapies and research in this area [12, 14]. The Foucauldian shift in the ethical discussion around diagnosis highlights that social assumptions can lead to further research in certain contexts and become materially effective. The examples presented confirm Foucault’s concept of epistemic conditionality in contemporary contexts.

Another aspect of epistemic conditionality highlighted by the Foucauldian perspective is the risk of blind spots within diagnostic categories. The struggle for recognition reveals that certain forms of suffering may fail to be acknowledged as diagnoses. One example is myocardial infarction, the symptoms of which can differ between women and men. In the past, however, medical research and clinical practice have focused primarily on the patterns of symptoms observed in men, which has led to underdiagnosis in women [9]. Although awareness of these differences has increased and is now reflected in medical training, women still face delays in diagnosis and treatment [36]. This demonstrates how male-centred norms can produce blind spots in both research and healthcare. Similar issues arise in pharmacology. For example, dosage recommendations for heart medicines are often geared towards men, whereas lower doses would be safer and more effective for women. Nevertheless, gender-specific dosage recommendations are not provided as standard. This perspective highlights the fact that certain forms of suffering are overlooked or misjudged. It shows that gender-specific stereotypes and norms can influence research questions and medical practices, and that gaps in knowledge regarding symptoms or therapeutic approaches for women arise [57].

These examples emphasise the importance of identifying forms of suffering that remain unnamed – whether due to a lack of recognition, limited research methods, or stereotypes that make it difficult to investigate them. Ultimately, for treatment methods to be developed and integrated into medical practice and terminology, it is essential that a condition is recognised as such [11, 25]. The clinical gaze can apprehend only what presents itself to it as a recognizable category, and for which it possesses the appropriate instruments of observation. These references and considerations open up the possibility of a deeper scientific-theoretical critique of these biomedical instruments and clinical practices. For ethical reflection in application, however, they emphasise the relevance for an awareness that this view can miss something, that it may not be all-encompassing.

The foregoing examples provide only a brief glimpse into the factors that currently shape the production of medical knowledge and diagnostic practices. Such factors can—and indeed must—be critically distinguished and reflected upon within concrete ethical debates on particular diagnoses. From Foucault’s perspective, it becomes evident that social forces play a decisive role in determining what is recognized as illness. Thus, the epistemic conditionality that Foucault repeatedly addresses in various works shows that the questions investigated in biomedical research and the resulting diagnoses are dependent on interests, value systems and linguistic and scientific possibilities. As discussed above, this observation does not imply that the resulting findings of biomedical research are false; however, they are not independent of the social sphere. The ethical discourse on diagnoses must account for this interplay between social dimensions and the construction of truth.

### Biopolitics of prevention

The Foucauldian turn by focusing on what precedes of the diagnostic moment reminds us in addition to the considerations above that the non-biomedical component of diagnosis always circulates within groups and depends on them. The impact of diagnosis on the patient depends on the norms, habits, and assumptions about illness held by groups and the public sphere. The diagnosed individual encounters pre-existing assumptions in her social environment. Examining the factors preceding the diagnostic moment reveals that assumptions and interests not only influence the development of biomedical categories but they also have an effect on the individual through collectives. This is due to the fact, that through collectives the regime of truth is formed and maintained [18]. Medical prevention and the promotion of health through specific behaviors can constitute part of such a regime of truth. In this way, the categories of desired and undesired behaviour can be linked to health and pathology in the public perception. Desired behavior such as health promoting behaviour can thus become the norm, while undesired behavior becomes pathology and is subject to prejudice. The result is a complex dynamic between biomedical knowledge and social desirability.

Through the Foucauldian shift, prevention and individual responsibility emerge as an effect of the connection between truth and power, highlighting their role as strategies for promoting social desirability [42, 50]. With regard to the second half of his work and the concept of biopower, Foucault shows explicitly that the stigma of illness intertwines with the appeal to avoid getting sick in the first place [24]. Thus, the social desirability associated with prevention, can link unhealthy behaviour with, for example, guilt, irresponsibility and expectations of prevention for individuals. Prevention thus offers “languages and techniques of selfunderstanding and self-mastery“ [49]. In this way, individuals are encouraged to adopt healthy habits and thus fulfil their responsibilities as rational, autonomous and self-regulating subjects. Failure to implement these preventive measures may then be stigmatised and even regarded, as irresponsible or a lack of autonomy.

These considerations do not call into question the valuable work of medical prevention but instead highlight that prevention itself carries a social dimension, particularly when illness occurs despite preventive measures. This risk demands ethical attention, since health-related personal responsibility often depends on material resources and, in cases of illness, can lead to the assignment of blame. Thus, considering the concept of biopower, the Foucauldian shift invites critical reflection on the assumptions surrounding responsibility and blame in the context of diagnosis and prevention. A Foucauldian approach is not limited to analysing the conditions that make certain forms of knowledge and diagnosis intelligible; it also examines how such diagnoses are socially valued and hierarchically situated within regimes of truth.

### Reframing diagnosis: a Foucauldian contribution

Applying Foucault’s work to the context of diagnosis raises a range of ethical questions that focus on the period prior to the diagnosis being made. On the one hand, it theoretically interrogates the emergence of diagnostic categories and the production of medical knowledge; on the other, it considers the social dynamics and collective assumptions about illness that shape individuals’ preventive behaviors before the onset of a disease. In summary Foucault is helping to focus on what precedes the application of diagnostic categories—what occurs before diagnosis—, to give attention to the beliefs of groups and economic or normative interests. The perspective of medical ethics presented at the beginning, which focuses on the effects of diagnosis, is changed and expanded by his perspective. However, the relevance of considering the effects of diagnosis is not questioned. Rather, a Foucauldian perspective proves valuable in tracing the origins of exclusion and stigma associated with diagnostic categories, particularly when the development of these categories is analyzed prior to their clinical application. This focus reveals that there might not be a domain of biomedical research that is shielded from contextual conditions. Such an approach should likewise constitute a central aim of ethical reflection.

Incorporating this shift into ethical research means that examining the period prior to diagnosis and the creation of a diagnostic category raises questions regarding the significance of a community’s beliefs and values that influence diagnoses. It requires a critical examination of which social standards must be met for a form of suffering to be recognised as a medical issue. Following Foucault, the question remains: How comprehensive is the clinical perspective when it comes to understanding human suffering, and which important aspects tend to be overlooked in the process?

These considerations are essential for a comprehensive ethical reflection on the handling and application of diagnostic categories in medicine. Beyond these questions, Foucault´s perspectives introduces additional thoughts for applied ethics. His approach leads to the question if diagnostic practices are at all times appropriate, necessary, and ethically mandatory. It interrogates the relationship between the implications of diagnoses, their claims to truth and their therapeutic potential. Social suffering associated with diagnoses can be understood in relation to the broader conditions that shape the status and legitimacy of diagnostic categories. In this way, the *before* and *after* of diagnostic processes are brought into conversation, thereby establishing a foundation for comprehensive reflection on these interconnected aspects.

An ethical approach to diagnoses thus requires a reconsideration of their status in practice—that is, evaluating the utility of such categorizations for individuals in light of their status as objective truths. The impact of diagnosis, including potential harm to social relationships and the psychological burden it may impose on individuals, can be weighed against two factors: the status of the diagnostic category as influenced by social conditions, and it´s therapeutic potential. By doing so, the Foucauldian shift examines in depth and with precision the risk that diagnosis may act as a rupture, dividing a life into a ‘before’ and an ‘after’ in a manner that is radical and abrupt [16]. This radicality, must be judiciously weighed against the capacity to heal and alleviate suffering, while remaining cognizant of the sociocultural context from which biomedical categories emerge.

## Conclusion

The paper addresses the complexity of medical diagnoses, which are not merely biomedical categories but also bear significance within the social sphere. It demonstrates that the social consequences of diagnoses rightly warrant attention in medical ethics research, as they entail risks for the patients such as stigmatization, social exclusion and treatment failure. An attempt is made to broaden the established medical ethics focus on the social impact of diagnoses through Foucault’s framework, illustrating that diagnoses are not merely imbued with social significance through their application. Rather, the Foucauldian perspective calls to mind that diagnoses are inherently shaped by interests, assumptions, and privileges in their creation and use and affect collectives and individuals even before they are applied. It is shown that this perspective does not diminish the status of scientific findings; rather, it ensures and safeguards their ethical application through a more comprehensive deliberation. Building on this foundation, the present work examines the field of theoretical and practical processes that precede the diagnostic moment, with particular attention to the influence of public interests on biomedical research and a focus on medical prevention. It also draws attention to potential blind spots, i.e. conditions that may go unrecognised and consequently remain without medical treatment and support, as well as the radical nature and risk of marginalisation that a diagnosis can entail. Taking these complex interrelations into account, questions for the theoretical reflection on medical diagnoses were formulated, leading to the conclusion that diagnostic practices must be evaluated for their appropriateness and utility - before, during and after their application.

## Data Availability

No datasets were generated or analysed during the current study.
